# Comparative Analysis of the Complete Mitochondrial Genomes for Development Application

**DOI:** 10.3389/fgene.2018.00651

**Published:** 2019-03-06

**Authors:** Nwobodo Alexander Kenechukwu, Man Li, Lei An, Miaomiao Cui, Cailin Wang, Aili Wang, Yulin Chen, Saijun Du, Chenyao Feng, Sijin Zhong, Yuying Gao, Xueyan Cao, Li Wang, Ezenwali Moses Obinna, Xinyu Mei, Yuanjian Song, Zongyun Li, Dashi Qi

**Affiliations:** ^1^Jiangsu Key Laboratory of Brain Disease Bioinformation, Xuzhou Medical University, Xuzhou, China; ^2^Department of Genetics, Research Center for Neurobiology, Xuzhou Medical University, Xuzhou, China; ^3^Huaihe Hospital of Henan University, Henan University College of Medicine, Kaifeng, China; ^4^Institute of Gastrointestinal Surgery and Translational Medicine, Tongji University School of Medicine, Shanghai, China; ^5^Department of Clinical, Xuzhou Medical University, Xuzhou, China; ^6^Department of Applied Biochemistry, Enugu State University of Science and Technology, Enugu, Nigeria; ^7^Interdisciplinary Research Center on Biology and Chemistry (IRCBC), Chinese Academy of Sciences, Shanghai, China; ^8^School of Life Sciences, Jiangsu Normal University, Xuzhou, China

**Keywords:** mitochondrial genome, *Serranochromis robustus*, *Buccochromis nototaenia*, development application, tRNA

## Abstract

This present research work reports the comparative analysis of the entire nucleotide sequence of mitochondrial genomes of *Serranochromis robustus* and *Buccochromis nototaenia* and phylogenetic analyses of their protein-coding genes in order to establish their phylogenetic relationship within Cichlids. The mitochondrial genomes of *S. robustus* and *B. nototaenia* are 16,583 and 16,580 base pairs long, respectively, including 13 protein-coding genes (PCGs), 2 ribosomal RNA genes, 22 transfer RNA genes, and one control region (D-loop) which is 888 and 887 base pairs long, respectively, showing the same gene order and identical number of gene or regions with other well-elucidated mitogenomes of Cichlids. However, with exception of cytochrome-c oxidase subunit-1 (*COX-1*) gene, all the identified PCGs were initiated by ATG-codons. Structurally, 11 tRNA genes in *B. nototaenia* species and 9 tRNA genes in *S. robustus* species, folded into typical clover-leaf secondary structure created by the regions of self-complementarity within tRNA. All the 22 tRNA genes in both species lack variable loop. Moreover, 28 genes which include 12-protein-coding genes are encoded on the H-strand and the remaining 9 genes including one protein-coding gene are encoded on the L-strand. Thirteen sequences of concatenated mitochondrial protein-coding genes were aligned using MUSCLE, and the phylogenetic analyses performed using maximum likelihood and Bayesian inference showed that *S. robustus* and *B. nototaenia* had a broad phylogenetic relationship. These results may be a useful tool in resolving higher-level relationships in organisms and a useful dataset for studying the evolution of the Cichlidae mitochondrial genome, since Cichlids are well-known model species in the study of evolutionary biology, because of their extreme morphological, biogeographical, parental care behavior for eggs and larvae and phylogenetic diversities.

## Introduction

*Serranochromis robustus* and *Buccochromis nototaenia* are essential species in commercial fisheries that belong to the family of Cichlidae; both are commonly found in tropical freshwater in estuaries in Lake Malawi, upper Shire River, Luongo River in the Congo, and Zambia. They respond promptly to environmental alterations and are both carnivorous and oviparous maternal mouthbrooder fish. *Buccochromis* consists of four different subspecies, *B. rhoadesii, B. spectalbilis, B. Lepturus*, and *B. nototaenia*, which stay at an average depth of 10 m in offshore sandy beaches. There are two subspecies of *serranchromis, S. robustus robustus* and *S. robustus jallae*. The latter is found in Cunene, Okavango, Kafue, upper and the middle Zambezi, Luangwa Luapula-Moero, Lualaba, and kassi-rivers and it has been transferred to Zimbabwe, Limpopo River and Natal, South Africa (Jellum, 1970; Kocher, 2004). The phylogenetic diversity of Cichlid fish, a vital tool in the study of evolutionary biology can be well-apprehended through proper investigation on mitochondrion, an evolutionary endosymbiont derived from bacteria (prokaryotes) (Sagan, 1967). The circular mitochondrial DNA (mtDNA) which can reproduce independent of its cell is an apparent cause of endosymbiosis. This is based on its close similarities with prokaryotes in their circular DNA, 70 ribosomes, 22 transfer RNAs, formyl-methionine initiating amino acid, and their susceptibility to tetracycline. The integral inner membrane proteins are synthesized from circular mtDNA, while proteins of the outer membrane of the mitochondria are translational products of nuclear mRNA. Since mitochondria are evolutionary endosymbionts that were derived from bacteria. The cellular injury will release mitochondria “damage-associated molecular patterns” DAMPs (formyl-peptides and circular mtDNA) into the circulation with functional critical immune consequences, and this is believed to be the link between trauma, inflammation, and systemic inflammatory response syndrome (SIRS) (Zhang et al., 2010). The mitochondrial genome of fish is a circular double-stranded molecule which is about 15 to 19 kb in length (Cui et al., 2017). Mitochondria play a central role in metabolism (Brand, 1997), including oxidative phosphorylation (Smeitink et al., 2001), apoptosis (Kroemer et al., 1998), diseases (Graeber and Muller, 1998), aging (Wei, 1998), and also different other biochemical functions.

However, because of the conserved characteristics of coding content, location on Y-chromosomal DNA, rapid evolution, and low levels of intermolecular genetic recombination, mitogenomes are biomarkers for molecular research in such areas as phylogenetic molecular evolution, population genetics, and evolutionary genomics (Bentzen et al., 1998; Boore, 1999). A typical mitochondrial genome contains typically two ribosomal RNA genes (s-rRNA and l-rRNA), 22 transfer RNA genes (tRNAs), 13 protein-coding genes (PCGs), and two typical non-coding control regions {control region (CR) and origin of the light strand (OL)} with regulatory elements essential for transcription and replication (Miya and Nishida, 1999). In some mitochondrial genomes the genes are located on both strands, whereas in others, all genes are transcribed from one strand (Ojala et al., 1981). Twelve of the 13 protein-coding genes are located on the H-strand, and only ND6 gene is located on the L-strand. The energy values of foods are harnessed in the form of ATP through coupling of energy-releasing activities of electron transport chain, proton pump, and oxidative phosphorylation (Cadenas and Davies, 2000). The electron transport chain has been proven to be “leaky.” The leaky nature of the electron transport chain results in the conversion of molecular oxygen into superoxide anion radical (${\text{O}}_{2}^{-}$) (www.ncbi.nlm.nih.gov). Dismutation of superoxide produces hydrogen peroxide (H_2_O_2_). Hydrogen peroxide may, in turn, be partially reduced to hydroxyl radical (HO^−^) or entirely reduced to water. These free radicals generated from mitochondria or other sites inside or outside the cell leads to cell (destruction) damage through mtDNA, RNA, and protein modifications and lipid peroxidation (Cline, 2012). Mutated mitochondrial DNA molecules accumulate with age. This age-dependent accumulation of mutated mtDNA (Lagouge and Larsson, 2013), can in principle be explained by two primary mechanisms, replication error, and unrepaired damage. First, it has been suggested that the massive mtDNA replication occurring during embryogenesis will result in replication errors due to the inherent error rate of the mitochondrial DNA polymerase. The mtDNA mutations formed during embryogenesis will be subjected to segregation and clonal expansion in postnatal life. Secondly, the alternative proposal states that damage caused by ROS, may overwhelm the repair machinery and result in accumulation of mutated mtDNA (Larsson, 2010; Lagouge and Larsson, 2013). Over the last 10 years, these properties of mtDNA namely; high mutation rate, fast evolutionary rate, and non-significant genetic recombination have made it universally accepted biomarker for determination of genetic diversity among species (Galtier et al., 2009). Cichlids are universal accepted species model in the study of phylogenetic biology due to their extreme diversified morphological and biogeographic traits, parental care behavior for eggs and larvae (Klett and Meyer, 2002; Kocher, 2004; Salzburger and Meyer, 2004; Seehausen, 2006; Turner, 2007; Kuraku and Meyer, 2008). Fish of this family are popularly remarked with two distinct features; a single opening of the nostrils and an interrupted lateral line (Hartvigsen and Halvorsen, 1994; Khuda-Bukhsh and Chakrabarti, 2000). As economically important fish, some Cichlids are widely used extensively in aquaculture for several reasons. They are good source of “white fish” and fish products, they lack small bones in the muscle, and some species can grow quite large, allowing for the production of value-added products like filets. Most essentially, they depend on the lower food chain (aquatic plants and plankton) reducing their cost of feeding (Pulling, 1991). in this study, we sequenced the complete mitochondrial genome of the two Cichlidae species and investigated the gene content and organization compared with other species. We also reconstructed phylogenetic tree based on PCG sequences for the purpose of analyzing the evolutionary relationships within Cichlids family. These results may provide more insight and a useful dataset for studying the evolution of the Cichlidae mitochondrial genome.

## Materials and Methods

### Sampling and DNA Extraction

The samples of *S. robustus* and *B. nototaenia*, collected from a trawler catch in the Southeast Arm of Lake Malawi (between latitudes 9° and 18°S, and longitudes 32° and 36°E) by the Welcome Trust Sanger Institute (SC) in their private collection, were not endangered or protected species according to the IUCN Red List. The circular mitochondrial genomic DNAs were extracted from dorsal muscle tissue samples using the Animal Tissue Genomic DNA Extraction Kit (SangonBiotech China) according to the manufacturer's instructions. Every protocol were performed in accordance with the international guidelines concerning the care and treatment of experimental animals. A known volume (15 ml) of 95% ethanol were used to preserve all the samples at −80°C until DNA extraction. Polymerase Chain Reaction was used to amplify the complete mitogenomes of the extracted DNA.

### Mitochondrial DNA Amplification and Sequencing

Eppendorf Thermal Cycler (Eppendorf, Germany) was used to perform polymerase chain reaction with a fix reaction mixture of 50 μl (microliter) consisting of 2 units of Taq DNA polymerase, 5 μl PCR buffer (Tiangen products, China), 2 μl template DNA (50 ng/ μl), 2 μl dNTP (0.4 mM), 4 μl primers (0.2 μM each), and 35 μl deionized/distilled water. After 3 min the reaction was denatured at 95°C, followed by 35 cycles of denaturation at 95°C for 30 s, annealing at 50°C for 30 s, and extension at 72°C for 1–5 min. All the PCR products were sequenced using the primer walking method with a 3730XL DNA Analyzer. The obtained sequences had 100% coverage of the PCR products.

### Sequence Editing and Analysis

All the reads were mapped to full mitochondrial genome reference sequences of *A. geoffreyi* (NC_028033) by using SOAPalingner/soap2 (V2.21). Then we assembled the roads which could map to the reference genome by SPAdes3 (V3.1.0) and got the circular mitochondrial genome. Additionally, the location of the 13 PCGs and the two rRNAs for each species were primarily identified through Dual Organellar Genome Annotator (DOGMA) (Wyman et al., 2004). The tRNA-scan-SE1.21 identified most of the transfer RNA (tRNA) genes from the website http://lowelab.ucsc.edu/tRNAscan-SE/, using the default search mode and the “Mito/chloroplast” source (Lowe and Eddy, 1997). To infer the secondary structures of tRNA molecules, we used a widely scientifically accepted comparative approach to correct for unusual pairings with RNA-editing mechanisms that are well-known in fish mitogenomes. The software RNA structure was used in drawing the secondary structure of tRNA genes (Mathews, 2014). The skewness of the nucleotide compositions were measured according to the following formulas: AT skew [(A – T) / (A + T)] and GC skew [(G – C)/ (G + C)] (Perna and Kocher, 1995). The full mitochondrial genomic DNA sequence of the *S. robustus* and the *B. nototaenia* were stored in the GenBank database with the following accession numbers, accession KX595333 and KX631426, respectively.

### Phylogenetic Analysis

To establish evolutionary relationships between *S. robustus* and *B. nototaenia* mitogenomes within the family Cichlidae the complete mitogenome sequence of *S. robustus* and *B. nototaenia* and 13 other species available in GenBank were used. Both amino acid and nucleotide sequences for each species of the 13 PCGs were aligned using default settings and concatenated, which were used for phylogenetic analysis through the Maximum Likelihood (ML) and Bayesian inference (BI) methods. Using raxmlGUI v 8.0.26 and MrBayes v 3.2.4, respectively (Ronquist et al., 2012; Silvestro and Michalak, 2012), which allowed different substitution models in individual partitions. Clustal X with default settings were used to align all the genes separately (Thompson et al., 2002). However, GTR + I + G was selected as the appropriate model for the nucleotide sequences by Modeltest 3.7 based on Akaike's information criterion (AIC) (Beier et al., 2004). MtArt + I + G + F were the appropriate model for the amino acid sequence dataset according to ProtTest 3.4 based on AIC (Abascal et al., 2005). The resulting phylogenetic trees were drawn in Molecular Evolutionary Genetics Analysis (MEGA) version 6.0 (Lewis et al., 1994).

## Results and Discussion

### Mitochondrial Genome Organization and Composition

**Figure 1:**
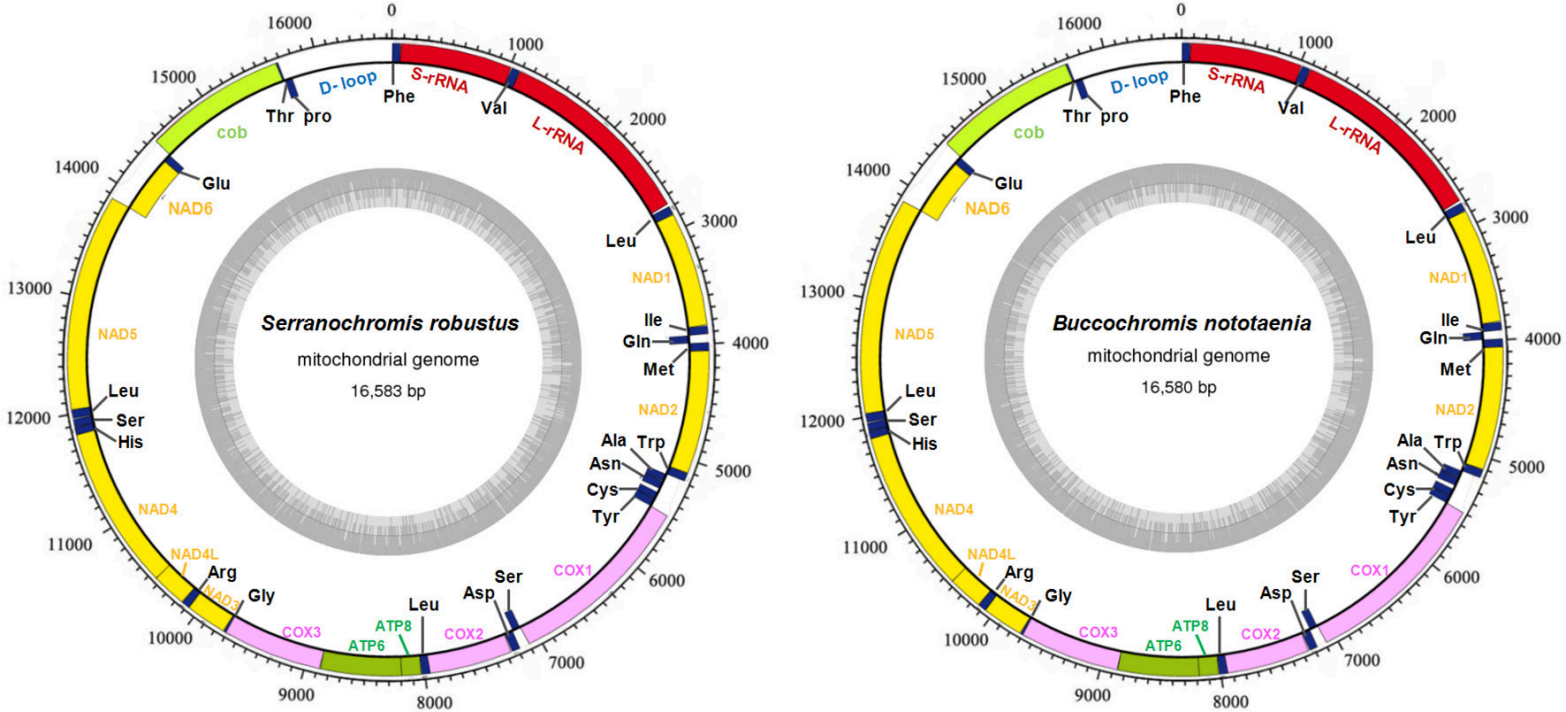
Circular map of the mitogenome of *S. robustus* and *B. nototaenia*. Genes encoded on the heavy or light strands are shown outside or inside the circular gene map, respectively, the size of the genome is 16,583 bp for *S. robustus* and 16,580 bp for *B. nototaenia*, where both contain the known 13 protein-coding genes of the respiratory chain (ND1, ND2, COX1, COX2, ATP8, ATP6, COX3, ND3, ND4L, ND4, ND5, ND6, and CYTB), 22 interspersed transfer RNA genes, 2 ribosomal RNA genes (12S and 16S rRNA) and one non-coding control region (D-loop region).

The structure of the mitochondrial genome of the newly sequenced *S. robustus* and *B. nototaenia* are similar to those of other cichlids characterized so far example., *F. rostatus*, and *A. geoffreyi* (Qi et al., 2016), they have the same types, number, and genomic features. The sequence data were deposited in GenBank under accession KX595333 and KX631426, respectively. The complete mitochondrial genomes were 16,583 bp long for *S. robustus* and 16,580 bp long for *B. nototaenia*, both were closed circular DNAs. The two species contain 13 protein-coding genes (*ATP*6, *ATP*8, Cytb, Cox1-3, ND1-6, and ND4L), 22 interspersed transfer RNA (tRNA) genes, 2 ribosomal RNA (rRNA) genes (s-rRNA and l-rRNA), and one control region (CR; also termed displacement loop region or D-Loop) (Table 1). Among these genes, 28 including 12 protein-coding genes are encoded on the H-strand and the remaining 9 genes including one protein-coding gene are encoded on the L-strand (ND6, Gln, Ala, Asn, Cys, Try, Ser, Glu, and Pro). The overall base composition of the mitochondrial genome is highly similar between these two species: A = 4,555 (27.47%), G = 2,599 (15.67%), T = 4,414 (26.62%) in *S. robustus* and A = 4,555 (27.47%), G = 2,624 (15.83%), T = 4,409 (26.59%) in *B. nototaenia*, whereas C = 4,993 (30.11%) in both species. However, the C content is relatively lower when compared with *P. managuensis* (31.0%) (Liu et al., 2016). An illustration of the complete mitochondrial genome of *S. robustus* and *B. nototaenia* is shown in Figure 1.

**Table 1:** Characteristic constituents of the mitochondrial genome organization of *S. robustus* and in *B. nototaenia*. H and L refer to the heavy and light strand, respectively.

**Feature**	**Strand**	***S. robustus***						***B. nototaenia***					
		**Position Start/End**	**Length**	**Codon Start/Stop**	**G+C%**	**GC Skew**	**RNAstructure energy**	**Position Start/End**	**Length**	**Codon Start/Stop**	**G+C%**	**GC Skew**	**RNAstructure energy**
tRNA-Phe	H	1/69	69				−14.8	1/69	69				−14.9
S–rRNA	H	70/1010	941					70/1010	941				
tRNA–Val	H	1013/1084	72				−17.7	1013/1084	72				−11.4
I–Rrna	H	1085/2760	1,676					1085/2766	1,682				
tRNA-Leu	H	2778/2851	74				−18.6	2776/2849	74				−18.6
Nadl	H	2852/3826	975	ATG/TAG	48.1	−0.40299		2850/3824	975	ATG/TAG	47.38	−0.4026	
tRNA–I1e	H	3830/3899	70				−17.9	3828/3897	70				−17.9
tRNA-Gln	L	3899/3969	71				−17.1	3897/3967	71				−17.1
tRNA-Met	H	3969/4037	69				−10.3	3967/4035	69				−10.3
Nad2	H	4038/5084	1,047	ATG/TAA	46.23	−0.50826		4036/5082	1,047	ATG/TAA	47.47	−0.49698	
tRNA-Trp	H	5084/5155	72				−27.2	5082/5153	72				−27.2
tRNA-Ala	L	5157/5225	69				−12.6	5155/5223	69				−12.6
tRNA-Asn	L	5227/5299	73				−21.2	5225/5297	73				−21.2
tRNA-Cys	L	5335/5401	67				−19.2	5333/5399	67				−19.2
tRNA-Tyr	L	5402/5471	70				−19.7	5400/5469	70				−20.2
Cox1	H	5473/7062	1,590	GTG/TAG	45.91	−0.21096		5471/7063	1,593	GTG/TAA	45.95	−0.20765	
tRNA-Ser	L	7066/7136	71				−17.7	7064/7134	71				−17.7
tRNA-Asp	H	7140/7212	73				−23.3	7138/7210	73				−23.3
Cox2	H	7218/7916	699	ATG/AGA	43.06	−0.26246		7216/7914	699	ATG/AGA	43.92	−0.27687	
tRNA-Leu	H	7909/7982	74				−14	7907/7980	74				−15
Atp8	H	7984/8151	168	ATG/TAA	44.64	−0.46667		7982/8149	168	ATA/TAA	44.64	−0.49333	
Atp6	H	8142/8825	684	ATG/TAA	48.54	−0.46988		8140/8823	684	ATG/TAA	47.66	−0.46012	
Cox3	H	8825/9664	840	ATG/TAA	46.07	−0.3075		8823/9662	840	ATG/TAA	46.67	−0.30102	
tRNA-Gly	H	9609/9680	72				−14.4	9607/9678	72				−14.4
Nad3	H	9681/10031	351	ATG/TAG	43.3	−0.39474		9679/10029	351	ATG/TAG	43.3	−0.39474	
tRNA-Arg	H	10030/10098	69				−10.7	10028/10096	69				−10.6
Nad41	H	10099/10395	297	ATG/TAA	48.82	−0.46207		10097/10393	297	ATG/TAA	48.48	−0.44444	
Nad4	H	10389/11774	1,386	ATG/AGA	46.52	−0.38605		10387/11772	1,386	ATG/AGA	46.54	−0.38605	
tRNA-His	H	11770/11838	69				−8.2	11768/11836	69				−9.6
tRNA-Ser	H	11839/11905	67				−14.8	11837/11903	67				−14.8
tRNA-Leu	H	11910/11982	73				−16.6	11908/11980	73				−16.4
Nad5	H	11983/13821	1,839	ATG/TAA	43.56	−0.42572		11981/13819	1,839	ATG/TAA	43.56	−0.42322	
Nad6	L	13818/14339	522	TTA/CAT	48.66	−0.55118		13816/14337	522	TTA/CAT	48.47	−0.54941	
tRNA-Glu	L	14340/14408	69				−10.5	14340/14408	69				−10.5
Cob	H	14413/15609	1,197	ATG/TAA	49.04	−0.36968		14411/15607	1,197	ATG/TAA	49.12	−0.36395	
tRNA-Thr	H	15554/15625	72				−20.7	15552/15623	72				−20.7
tRNA-Pro	L	15626/15695	70				−24.9	15624/15693	70				−22.3
D-loop	H	15696/16583	888					15694/16580	887				

### Protein Coding Genes

Protein-coding genes (PCGs) of the mitochondrial genome in *S. robustus* and *B. nototaenia* include 7 NDH dehydrogenase subunits (NAD1-6, NAD4l), 3 cytochrome c oxidase subunits (Cox1-3), 2 *ATP*ase subunits (*ATP*6, *ATP*8), and one cytochrome b gene (Cob), ranging in size from 168 bp (*ATP*ase8) to 1,839 bp (ND5). Frequently methionine (ATG) is the start codon for most PCGs genes, except for cox1, which utilizes GTG; this is an accepted canonical mitochondrial start codon for vertebrate mitogenomes (Yue et al., 2006; Yan et al., 2016; Yang et al., 2016). The termination codons of the 13 PCG can be classified into distinct types, the stop codons (TAA, AGA, CAT, TTA, and TAG) are utilized in these PCGs, seven PCGs (nd2, *atp*8, *atp*6, cox3, nad4l, nd5, cob) are terminated with the typical stop codon TAA, while two PCGs (ND1, ND2) has, (COX2, ND4) has AGA stop codon and (ND6) has stop codon in both species and (COX1) has stop codon in *S. robustus*, TAA in *B. nototaenia*. The detection of various stop codon types is common among vertebrate mitochondrial genome, and TAA stop codon appears via post-transcription polyadenylation (Ojala et al., 1981). Twelve of the 13 PCGs are encoded on H-stand, whereas only the nd6 gene is encoded on the L-strand (Table 1). Four reading-frame overlaps were observed in the two species. In *S. robustus*, 10 nucleotides overlap between ATP8 and ATP6, seven nucleotides overlap between ND4L and ND4, four nucleotides between ND5 and ND6 (opposite strands), and one nucleotide overlap between *ATP*6 and COX3. The same overlaps were also observed in *B. nototaenia*. The overlapping of protein-coding genes suggests partial sharing of transcripts among neighboring coding regions, which is commonly found in bony fishes (Montoya et al., 1983).

The total nucleotide length of the 13 PCGs is 11,595 bp long for *S. robustus*, accounts for 69. 92% of the whole lengths, and 11, 598 bp long for *B. nototaenia* accounting for 69.95% of the whole length. The AT and GC skew values of the PCGs of the two species are shown in Table 2. The A+T base composition is 54.22% for *S. robustus* and 54.07% for *B. nototaenia*, respectively. These values are higher than the G+C base compositions (45.78% in *S. robustus* and 45.93% in *B. nototaenia*). Additionally, the AT skew (0.0181) for the *S. robustus* mitogenome and (0.0163) *B. nototaenia* mitogenome are sliga greater occurrence of As to Ts, and its GC skew (−0.3153) and (−0.3109), respectively are negative, indicating a higher content of Cs than Gs. Among 13 PCGs examined in both species, the length of ND5 gene (1,839 bp) is the longest, whereas the shortest is *ATP*8 gene (168 bp) (Table 1). Both species contain nucleotide G least frequently in the third codon position. Our result indicates that more Ts and Cs are present in most PCGs, which is consistent with most previous observations (Hwang et al., 2013).

**Table 2:** List of species used in this study and their corresponding accession numbers included in phylogenetic and comparative analyses.

**Species**	**Kingdom**	**Phylum**	**Class**	**Order**	**Family**	**Genus**	**NCBI ID**
*Amblyraja radiata*	Metazoa	Chordata	Chondrichthyes	Rajiformes	Rajidae	Amblyraja	NC_000893
*Halocynthia roretzi*	Metazoa	Chordata	Ascidiacea	Stolidobranchia	Pyuridae	Halocynthia	NC_002177
*Huso dauricus*	Metazoa	Chordata	Actinopteri	Acipenseriformes	Acipenseridae	Huso	NC_023837
*Huso huso*	Metazoa	Chordata	Actinopteri	Acipenseriformes	Acipenseridae	Huso	NC_005252
*Hypoatherina tsurugae*	Metazoa	Chordata	Actinopteri	Atheriniformes	Atherinidae	Hypoatherina	NC_004386
*Menidia menidia*	Metazoa	Chordata	Actinopteri	Atheriniformes	Atherinopsidae	Menidia	NC_011174
*Alticorpus geoffreyi*	Metazoa	Chordata	Actinopteri	Cichliformes	Cichlidae	Alticorpus	NC_028033
*Copadichromis quadrimaculatus*	Metazoa	Chordata	Actinopteri	Cichliformes	Cichlidae	Copadichromis	KX272653
*Coptodon zillii*	Metazoa	Chordata	Actinopteri	Cichliformes	Cichlidae	Coptodon	NC_026110
*Fossorochromis rostratus*	Metazoa	Chordata	Actinopteri	Cichliformes	Cichlidae	Fossorochromis	NC_028089
*Hemitilapia oxyrhyncha*	Metazoa	Chordata	Actinopteri	Cichliformes	Cichlidae	Hemitilapia	KX594381
*Lethrinops lethrinus*	Metazoa	Chordata	Actinopteri	Cichliformes	Cichlidae	Lethrinops	KX595334
*Placidochromis longimanus*	Metazoa	Chordata	Actinopteri	Cichliformes	Cichlidae	Placidochromis	NC_028156
*Serranochromis robustus*	Metazoa	Chordata	Actinopteri	Cichliformes	Cichlidae	Serranochromis	KX595333
*Buccochromis nototaenia*	Metazoa	Chordata	Actinopteri	Cichliformes	Cichlidae	Buccochromis	KX631426

### Transfer RNA (tRNA) and Ribosomal RNA (rRNA)

**Figure 2:**
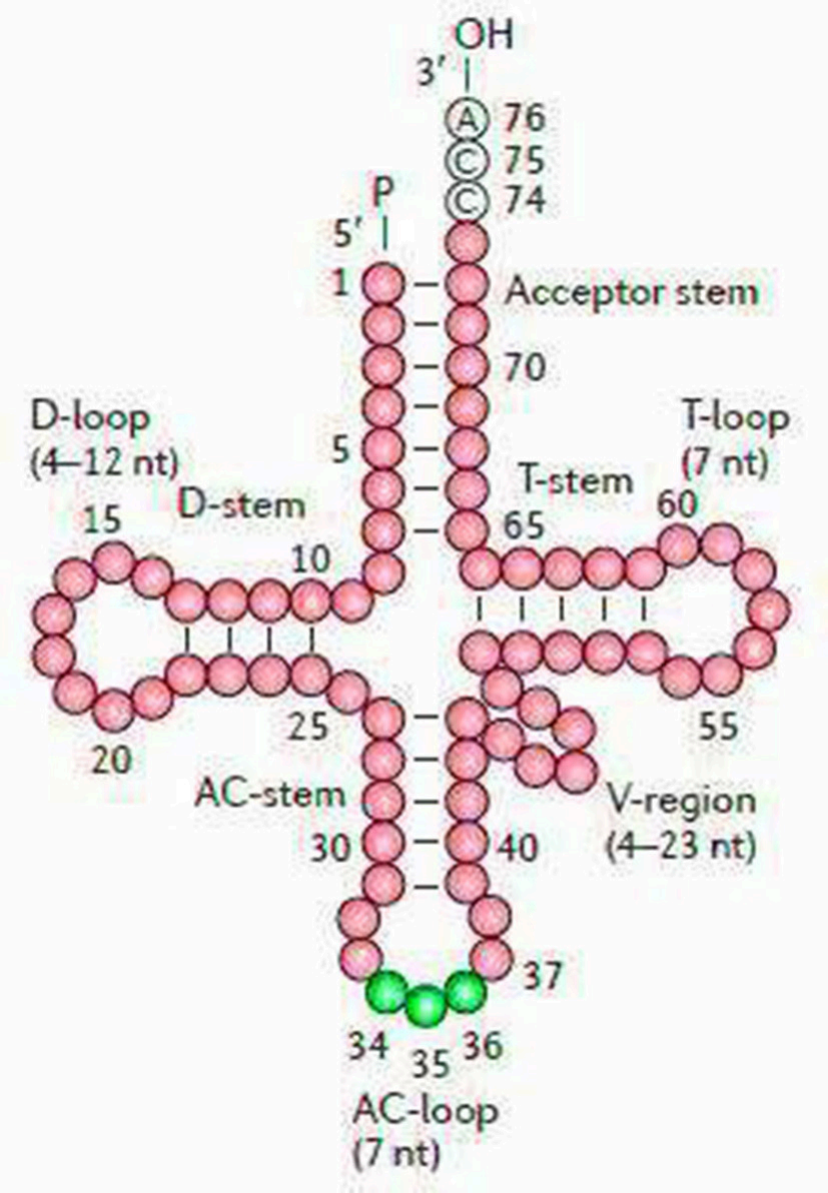
Clover leaf-like structure of tRNA. The tRNA possess the acceptor arm (7 nt), D-arm (3–4 nt), D-loop (4–12 nt), anti-codon arm (5 nt), anti-codon loop (7 nt), variable region (4–23 nt), 9-arm (5 nt), and 9-loop (7 nt). The D-arm, D-loop, and variable region possess variable number of nucleotides whereas the nucleotide number in the acceptor arm, anti-codon arm, anti-codon loop, 9-arm, and 9-loop is always constant.

**Figure 3:**
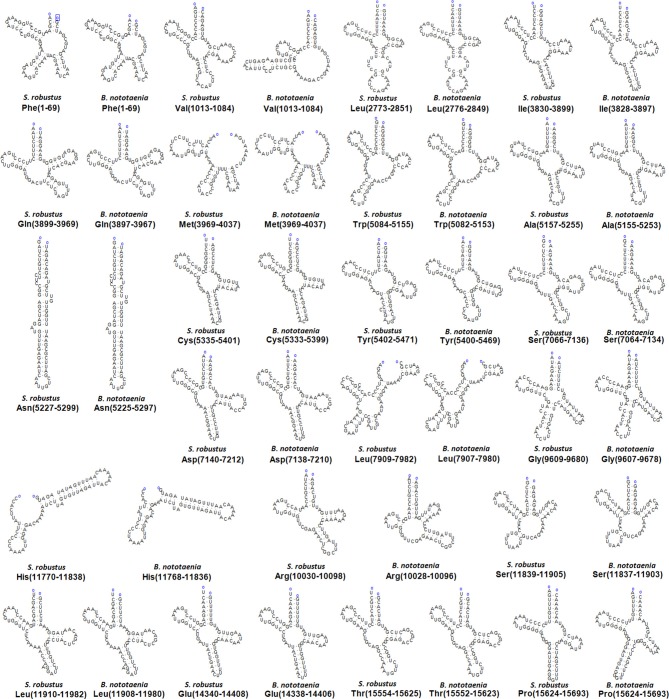
Secondary structures for the tRNA genes in the *S. robustus* and *B. nototania* mitogenome.

The secondary structures of 22 tRNA genes (typical clover-leaf secondary structure, including three for Leucine, two for Serine and one for each of the other amino acids) in the two fish mitogenomes are showed in Figures 2 and 3. The H-strand encodes Fourteen tRNAs, and the remaining 8 tRNAs are encoded by the L-strand (Table 1). All tRNAs varied in size from 67 bp (tRNA^cys^) to 74 bp (tRNA^lue^) in both species. This tRNA genomic, the molecular structural design is similar in most fish species ever examined such as *L. microptera* and *C. kumu* (Cui et al., 2017). Eleven tRNA genes in *B. nototaenia* species namely; {Ser (7064-7134), Gly (9607-9678), Arg (10028-10096), Phe (1-69), Trp (5082-5153), Gln (3897-3967), Ala (5155-5253), and Tyr (5400-5469)} and nine tRNA genes in *S. robustus* species namely; {Ser (7066-7136), Gly (9609-9680), Arg (10030-10098), Trp (5084-5155), Tyr (5402-5471), Gln (3899-3969), Ala (5157-5255), Leu (11910-11982), and Val (1013-1084)}, folded into typical clover-leaf secondary structures created by the regions of self-complementarity within tRNA (Figure 2). The data obtained further indicate that all the 22 tRNA genes in both species lack variable loop (Mohanta et al., 2017). The absence of D-arm (D-stem and D-loop) in the secondary structure of these transfer RNA {His (11768-11836), Leu (7907-7980), Asn and (5225-5297)} and [His (11770-11838), and Asn (5227-5299)] in *B. nototaenia* and *S. robustus* respectively, invariably altered their recognition potentials (Hardt et al., 1993). The formation of tRNA ribosomal complex by Val (1013-1084) in *B. nototaenia* and Asn (5227-5299) and Leu (7909-7982) in *S. robustus* during protein biosynthesis and translation will be greatly hindered because of the absence T-arm (T-stem and T-loop) in their secondary structures, since it serves as specialized recognition site (region) in the ribosome. Organisms with tRNA lacking T-loop exhibit a much lower level of aminoacylation and EF-TU-binding than in organisms which have the native tRNA (Griffith et al., 1999).

**Figure 4:**
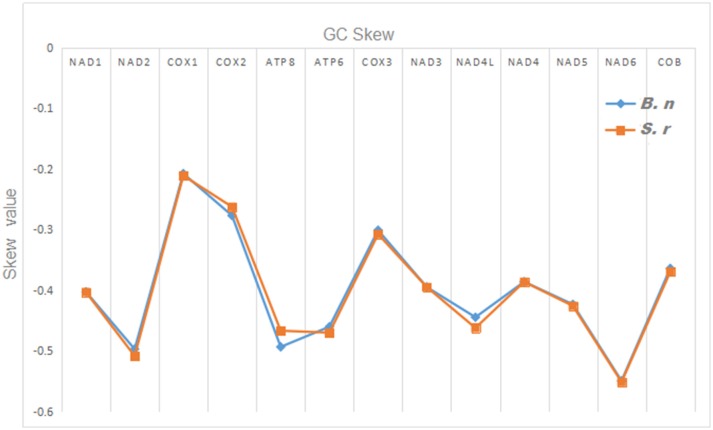
Graphical illustration showing the GC skew in the PCGs of the mitochondrial genome of *S. robustus* (S.r) and *B. nototaenia* (B.n).

**Figure 5:**
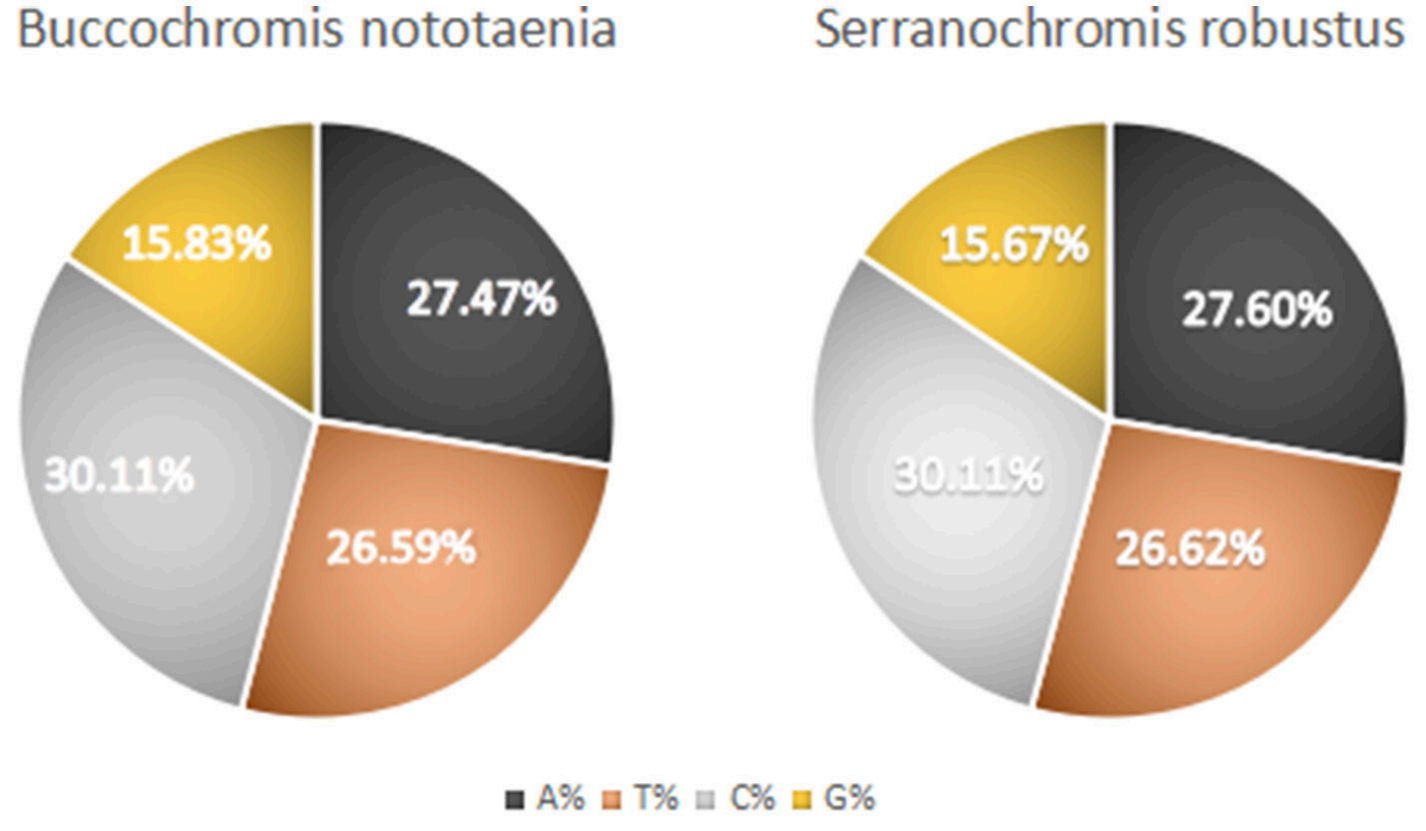
Base composition of the mitochondrial genome.

**Figure 6:**
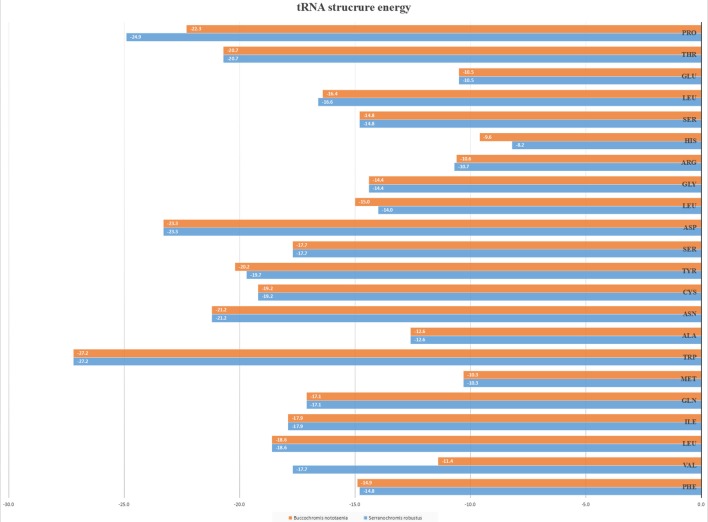
RNA structure energy strand.

However, the presence of unpaired 3 bases in the T-loops of {Pro (15624-15693), Cys (5333-5399), and Leu (2776-2849)} and {Cys (5335-5401), and Leu (2778-2851)} in both *B. nototaenia* and *S. robustus*, respectively, shows high degree of instability in their T-loops, since the optimal loop length of stability of the T-loop is 7 base pair long (Mohanta et al., 2017). The absence of acceptor's arm in the secondary structures of these transfer RNAs {His (11768-11836) and Met (3967-4035)} and {His (11770-11838), Leu (7909-7982), and Met (3969-4037)} in *B. nototeania* and *S. robustus*, respectively and Phe (1-69) with short acceptor arm of 2-base long in *S. rubustus* may affect their aminoacylation (Schimmel et al., 1993). The presence of unpaired 3-bases in the Anticodon-loops of {Glu (14338-14406), Asp (7138-7210), Pro (15624-15693), Ile (3828-3897), and Cys (5333-5399)} and {Glu(14340-14408), Asp (7140-7212), Pro (15626-15695), Ile (3830-3899), and Cys (5335-5401)} in both *B.nototaenia* and *S. robustus*, respectively show the degree of instability in their anticodon-loops, since the optimal loop length for stability of the Anticodon loop is 5 base pair long (Mohanta et al., 2017). The presence of two unusual tRNAs secondary structures, one with 4-loops and another without the T-arm and D loops {Leu (7907-7980), and Asn (5225-5297)} and {Leu (7909-7982), and Asn (5227-5299)} were identified in both *B. nototaenia* and *S. robustus*, respectively. The average A+T content of tRNA is 54.07% for *B. nototaenia* and 54.22% for *S. robustus* (Figures 4, 5). The mitochondrial genomes of *S. robustus* and *B. nototaenia* contain two subunits of ribosomal RNA, a small one (s-rRNA) and a large one (l-rRNA) (Figure 6). The two subunits were encoded in the same H strand, like other typical fishes and are separated by tRNA^val^. The length of the s-rRNA gene is 941 bp long in the two species; the length of the l-rRNA gene is 1,676 bp long for *S. robustus* and 1,682 bp long for *B. nototaenia*.

### Non-coding Regions

The non-coding regions in the mitochondrial genomes of *S. robustus* and *B. nototaenia* were flanked by tRNA^pro^ and tRNA^thr^ genes (Table 1). This present observation is quite different from most typical mitogenome, in which non-coding (D-loop) region were located between tRNA^pro^ and tRNA^phe^ genes (Lee et al., 1995; Yue et al., 2006). In this present study, the D-loop control regions were determined to be 888 and 887 bp in length for *S. robustus* and *B. nototaenia*, respectively. The non-coding region of both species is in agreement with observations in other species, with the D-Loop region divided into three primary domains. The first domain is hypervariable and consists of a termination-associated sequence (TAS), the second domain is the central conserved region, and the third domain comprises of three conserved blocks (CSB1, CSB2, and CSB3) (Kartavtsev et al., 2007).

### Phylogenetic Analysis

**Figure 7:**
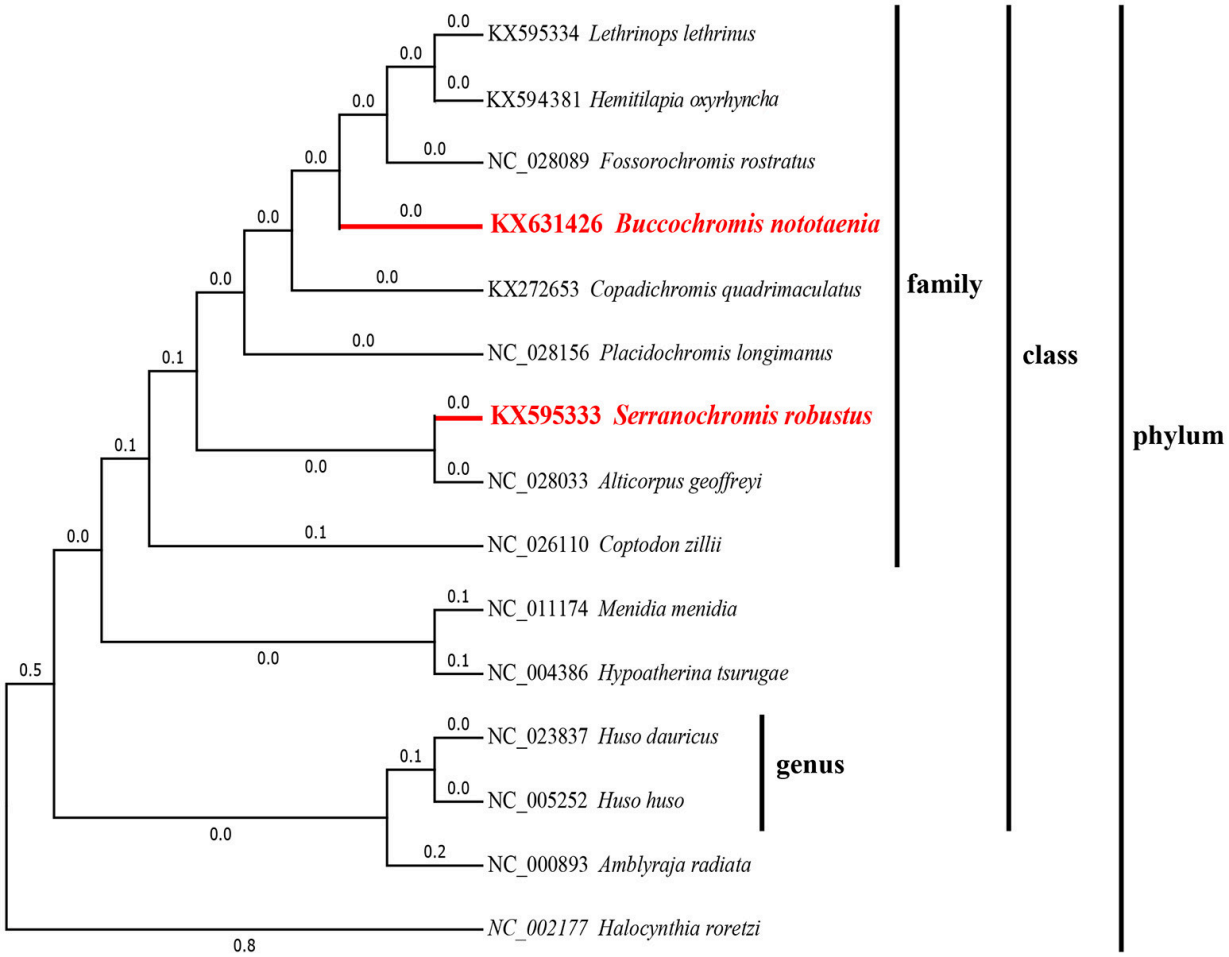
Phylogenetic trees inferred from amino acid and nucleotide sequences of 13 PCGs of the mitogenome. The phylogenetic analyses were conducted with maximum likelihood (ML) and Bayesian inference (BI). The numbers in front of the species are GenBank accession numbers.

To confirm the evolutionary position of *S. robustus* and *B. nototaenia*, we built a phylogenetic tree of 15 species of Cichlidae using published mitogenomes based on the concatenated nucleotide alignment of 13 PCGs via BI and ML methods (Figure 7 and Table 3). The results provide an excellent support for the monophyly of each family. This topology is mainly consistent with previously reported phylogenetic studies (Kartavtsev et al., 2007; Chen et al., 2017). The phylogenetic analysis using 13 concatenated mitochondrial protein-coding genes indicates that *S. robustus* and *B. nototaenia* had a broad phylogenetic relationship. However, in comparison with other 15 species of phylum chordate, {(*L. lethrinus, H. oxyrhyncha, F. rostratus, B. nototaenia, C. quadrimaculatus, P. longimanus, S. robustus, A. geoffreyi*, and *C. zilli)*} clustered in family Cichlidae, *S. robustus* and *A. geoffreyi* formed an independent monophyletic clade; therefore, the relationship between *S. robustus* and *A. geoffreyi* calls for further investigation. In further comparison with other established mitogenomes of *C. quadrimaculatus* and *P. longimanus*, our findings showed a close relationship between *B. nototaenia, C. quadrimaculatus*, and *P. longimanus* and a lineage of *S. robustus* and *A. geoffreyi* relatively distinct from *B. nototaenia*. This result is concordant with the evolutionary relationships inferred based on phylogenetic analysis.

**Table 3:** Base composition of AT and GC skew of the mitochondrial genome.

**Species**	**Length(bp)**	**A**	**T**	**C**	**G**	**A%**	**T%**	**C%**	**G%**	**A+T%**	**G+C%**	**AT skew**	**GC skew**
*Serranochromis robustus*	16,583	4577	4414	4993	2599	27.60	26.62	30.11	15.67	54.22	45.78	0.0181	−0.315331928
*Buccochromis nototaenia*	16,580	4555	4409	4992	2624	27.47	26.59	30.11	15.83	54.07	45.93	0.0163	−0.31092437

## Conclusion

The mitogenomes sequences of the two species (*S. robustus* and *B. nototaenia*) from the family Cichlidae were determined and compared alongside with those of other Chordata Species. Their complete mitogenomes indicate typical circular molecules and had similar genome organization and structure as those found in other Cichlids species. The length of the mitogenome sequences of *S. robustus* and *B. nototaenia* were 16,583 and 16,580 bp, respectively. Each mitogenome consists of a typical structure of 13 PCGs, 2 rRNAs, 22 tRNA genes, and one non-coding region. Similar to other vertebrate mitogenomes, most of the PCGs utilized as their initiation codons, except for cox1 which utilizes GTG. Additionally, 11 tRNA genes in *B. nototaenia* and 9 tRNA genes in *S. robustus* species, folded correctly into typical clover-leaf secondary structures created by the regions of self-complementarity with the exception of {His (11768-11836) and Met (3967-4035)} and {His (11770-11838), Leu (7909-7982), and Met (3969-4037)} in *B. nototeania* and *S. robustus*, respectively that lacked the acceptor arm and Phe (1-69) with short acceptor arm of 2-base long in *S. rubustus*. The presence of two unusual tRNAs secondary structures, one with 4-loops and another without the T-arm and D-loop {Leu (7907-7980), and Asn (5225-5297)} and {Leu (7909-7982), and Asn (5227-5299)} were identified in both *B. nototaenia* and *S. robustus*, respectively. All the 22 tRNA genes in both species lacked variable loop. The phylogenetic analysis using 13 concatenated mitochondrial protein-coding genes indicates that *S. robustus* and *B. nototaenia* had wide phylogenetic relationship. Comparison with other established mitogenomes suggests a close relationship between *B. nototaenia, C. quadrimaculatus*, and *P. longimanus* and a lineage of *S. robustus* and *A. geoffreyi* relatively distinct from *B. nototaenia*.

## Author Contributions

XM, ZL, YS, and DQ conceived and designed the study. MC, AW, LW, NA, CW, ML, MM, and LA conducted the molecular work and data analysis. NA drafted the manuscript. YC, SD, CF, AL, SZ, XC, and YG prepared all figures and tables. ML and LA performed the phylogenetic analyses. EO and ZL analyzed the tRNA and contributed in drafting the manuscript.

### Conflict of Interest Statement

The authors declare that the research was conducted in the absence of any commercial or financial relationships that could be construed as a potential conflict of interest.

## Abbreviations

PCGs: protein-coding genes
A: adenine
T: thymine
G: guanine
C: cytosine
ML: maximum likelihood
BI: Bayesian inference
BP: Base pair
mtDNA: mitochondrial DNA
*Atp*6 and Atp8: genes for the *ATP*ase subunits 6 and 8
Cox1-cox3: genes for cytochrome C oxidase subunits I–III
Nad1–nad6 and nad4L: genes for NADH dehydrogenase subunits 1–6 and 4L
rRNA: ribosomal RNA genes subunit
l-rRNA (large): rRNA subunit
s-rRNA: (small)
tRNAx: transfer RNA, where X is replaced by three letters amino acid code of the corresponding amino acid.
